# Folic Acid Attenuates Contrast-Induced Nephropathy in Patients With Hyperhomocysteinemia Undergoing Coronary Catheterization: A Randomized Controlled Trial

**DOI:** 10.3389/fcvm.2021.707328

**Published:** 2021-10-01

**Authors:** Long Peng, Xing Shui, Fang Tan, Zexiong Li, Yesheng Ling, Bingyuan Wu, Lin Chen, Suhua Li, Hui Peng

**Affiliations:** ^1^Department of Cardiovascular Medicine, The Third Affiliated Hospital, Sun Yat-sen University, Guangzhou, China; ^2^Department of Anaesthesiology, The Seventh Affiliated Hospital of Sun Yat-sen University, Shenzhen, China; ^3^Department of Anaesthesiology, The Third Affiliated Hospital, Sun Yat-sen University, Guangzhou, China; ^4^Department of Cardiovascular Medicine, Jieyang People's Hospital, Jieyang, China; ^5^Nephrology Division, The Third Affiliated Hospital, Sun Yat-sen University, Guangzhou, China

**Keywords:** contrast-induced nephropathy, folic acid, hyperhomocysteinemia, coronary catheterization, acute kidney injury

## Abstract

**Background:** Hyperhomocysteinemia is a risk factor for contrast-induced nephropathy. Folic acid can attenuate such nephropathies in rats. The protective effect of folic acid against contrast-induced nephropathy has not been studied in humans. We aimed to investigate the effect of folic acid on the incidence of contrast-induced nephropathy (CIN) after coronary catheterization in patients with hyperhomocysteinemia.

**Methods:** This was a single-center, prospective, double-blind, randomized controlled trial (ClinicalTrials.gov, NCT02444013). In total, 412 patients (mean age: 65 ± 12 years, 268 male) with plasma homocysteine ≥15 μM, who underwent coronary arteriography (CAG) or percutaneous coronary intervention (PCI) from May 2015 to August 2018, were enrolled. Patients were randomly assigned to two groups: a treatment group (*n* = 203), taking 5 mg of folic acid (orally, three times/day) immediately after enrollment and for 72 h after operation, and a control group (*n* = 209), taking placebo. Contrast-induced nephropathy was defined as an increase in serum creatinine of >25% or 44 μM within 48 or 72 h after contrast medium administration.

**Results:** In total, 50 (12%) patients developed CIN after 48 h after catheterization, including 16 (8%) in the treatment group and 34 (16%) in the control group (*P* = 0.009). Meanwhile, 53 (13%) patients developed CIN after 72 h of CAG/PCI, including 18 (9%) in the treatment group and 35 (17%) in the control group (*P* = 0.017). The incidence of contrast-induced nephropathy in the treatment group was lower than that in the control group (*P* = 0.017). Logistic regression analysis confirmed that administration of folic acid was a protective factor against contrast-induced nephropathy (RD = 0.0788, 95%CI: 0.0105–0.1469, *P* = 0.019). We found no serious adverse events associated with folic acid. No death or hemodialysis occurred in either group.

**Conclusions:** Perioperative administration of folic acid attenuates the incidence of contrast-induced nephropathy after coronary catheterization in patients with hyperhomocysteinemia.

**Clinical Trial Registration:** ClinicalTrials.gov, identifier [NCT02444013].

## Introduction

Contrast-induced nephropathy (CIN) is a common complication when using intravenous iodinated contrast. It is the third most common cause of hospital-acquired acute kidney injury, accounting for 11% of cases (1), second only to decreased renal perfusion and nephrotoxic medication (2). The overall incidence of CIN ranges from 3 to 7% but can be as high as 50% in patients with moderate to advanced chronic kidney disease (CKD) (3). CIN is associated with prolonged hospital stays, as well as an increased risk of CKD, cardiovascular events, and death (4). It has been reported that 49% of CINs occur after either coronary arteriography (CAG) or percutaneous coronary intervention (PCI) (5). Therefore, investigating the potential means to reduce the risk of CIN in patients undergoing CAG or PCI is warranted.

The pathogenesis of CIN has been explained by combinations of various mechanisms, such as renal vasoconstriction, increased oxidative stress, and impaired endothelial function, as well as renal tubular-cell cytotoxicity and apoptosis (6, 7). Homocysteine (Hcy), a sulfhydryl-containing amino acid, is closely associated with a risk of renal and cardiovascular diseases (8–10); hyperhomocysteinemia (HHcy) is known to induce oxidative stress, endothelial dysfunction, apoptosis, and thrombosis (9, 11). From previous studies, including our own (12), HHcy is a known independent risk factor for CIN (13).

Folic acid, an important dietary determinant of Hcy, exerts antioxidative and anti-apoptotic effects, and improves endothelial function; therefore, it plays a vital role in the management of cardiovascular diseases associated with HHcy (14, 15). A previous study revealed that folic acid could attenuate CIN in rats (16). However, the protective effect of folic acid against CIN has not been studied in humans. In this study, we aimed to investigate the effect of folic acid on the incidence of CIN after CAG or PCI in patients with HHcy.

## Materials and Methods

### Study Population

This was a single-center, prospective, double-blind, randomized controlled trial, registered on May 14, 2015 (ClinicalTrials.gov, NCT02444013). Patients with HHcy, scheduled to undergo CAG or PCI, were prospectively enrolled, after screening for eligibility, from May 2015 to August 2018, at the Department of Cardiovascular Medicine, the Third Affiliated Hospital, Sun Yat-sen University. The inclusion criteria were an age ≥18 years and a fasting plasma Hcy concentration ≥15 μM. The exclusion criteria were as follows: pregnancy; use of folic acid, vitamin B12, contrast agent, or nephrotoxic drugs within 14 days before enrollment; allergy to iodine-containing contrast medium; and end-stage renal failure. All procedures were conducted in accordance with the tenets of the Declaration of Helsinki, and all participants provided written informed consent before being enrolled in the study. This study was approved by the institutional review board of the Third Affiliated Hospital, Sun Yat-sen University (IRB: 20150216).

### Randomization and Masking

Patients were randomly assigned (1:1) to the group receiving oral 5-mg folic acid or the group receiving matching placebo three times/day. Patients were allocated to the two treatment arms by central computer allocation using simple randomization with no stratification factors. A random number table was generated by one researcher, and another researcher put the random number and group number into opaque envelopes of the same size and color. Randomization was performed by a third investigator. Random generation, concealment, and assignment were performed by three different investigators, none of whom participated in the subsequent part of the intervention program. All doctors and subjects were blinded to the allocation of drug administration, as a double-blinding method was used for the allocation.

### Procedures

As protection against the intravenous contrast medium, all patients received prophylactic hydration with 0.9% NaCl solution (1–2 mL/kg/h, intravenous), 6 h before and 6 h after contrast administration. Random number tables were used to assign patients to two groups: a treatment group, taking 5 mg of folic acid (orally, three times a day) immediately after enrollment, and a control group, taking a placebo at the same intervals.

Blood samples were collected at baseline and analyzed on a Hitachi 7180 clinical analyzer (Hitachi High-Tech Corp, Japan) for the evaluation of blood urea nitrogen, uric acid, blood lipids, cystatin C, and fasting glucose. Hemoglobin was measured using the cyanmethemoglobin method. Hemoglobin A1C was measured, *via* the D-10 Hemoglobin Testing System (Bio-Rad Laboratories, Inc., Hercules, CA, USA), using high-performance liquid chromatography. Plasma Hcy was measured on the ADVIA Centaur (Siemens Healthineers AG, Erlangen, Germany) at baseline, as well as the day before and 72 h after CAG/PCI. Serum creatinine (Scr) concentration was estimated using the sarcosine oxidase enzymatic method at baseline, the day before CAG/PCI, as well as at 24, 48, and 72 h after CAG/PCI. The estimated glomerular filtration rate (eGFR) was calculated using the equation developed by the Modification of Diet in Renal Disease study group (17).

Cardiac catheterization was performed in accordance with standard clinical practice, *via* a femoral or radial approach. Coronary angiography was routinely performed using the Judkins technique. Significant coronary artery disease was defined as >50% coronary stenosis in at least one vessel. The contrast type and dose were decided by two experienced interventional cardiologists based on operative requirements.

The primary outcome measure was the rate of occurrence of CIN. According to previous studies, CIN can be defined as either 48 or 72 h (18, 19). Therefore, in order to reduce the risk of obtaining different results due to the different criteria, we revised the registration scheme at the beginning of study, and used both CIN criteria for analysis. Thus, CIN was considered as an increase in Scr concentration of more than 25% or 44 μM within 48 and 72 h after contrast medium administration, without evidence of other causes. The secondary outcome measures were major in-hospital clinical events with 72 h, including: (1) bleeding, defined as a reduced hemoglobin level ≥20 g/L; (2) all-cause death; (3) dialysis or hemofiltration due to symptoms or signs of uremic syndrome or management of refractory hypervolemia, hyperkalemia, or acidosis; (4) worsening heart failure, defined as a deteriorated NYHA functional class; and (5) intensive care unit (ICU) admission. The safety outcome was measured by assessment of adverse reactions, such as nausea, decreased appetite, insomnia, and allergic reaction. In addition, we assessed the serial changes in serum creatinine and Hcy concentration.

### Statistical Analysis

Statistical analysis was performed using IBM SPSS Statistics for Windows version 22.0 (IBM Corp., Armonk, NY, USA).

We designed this study to assess the superiority of folic acid over the standard approach to reduce CIN in patients with hyperhomocysteinemia undergoing coronary catheterization. We calculated the necessary sample size on the basis of previous trial data suggesting that 15.4% of the control group and 6.34% of folic acid treatment group would develop CIN. We also estimated that 10% of patients would be lost to follow-up, based on previous CIN prevention trials enrolling patients undergoing cardiac procedures (20). On the basis of these assumptions, a chi-squared analysis suggested that 404 patients would be needed to detect a statistically significant difference, with 80% power and a two-sided α of 0.05.

Intention-to-treat (ITT) analysis was primarily used for this trial including all participants who received folic acid or placebo and underwent coronary angiography. Continuous variables are presented as mean ± standard deviation values, whereas categorical variables are presented as frequencies and percentages. Continuous variables were tested for a normal distribution using the Shapiro–Wilk test. Normally distributed variables were compared using two-tailed, independent *t*-tests. Non-normally distributed variables were compared using the two-tailed Mann–Whitney *U*-test. Repeated measures variables were analyzed using two-way repeated measures ANOVA and a linear mixed model to analyze changes. Categorical data were compared between the groups using Pearson's chi-square and Fisher's exact tests. Univariate logistic regression analyses were used to determine independent predictors of CIN. We used logistic regression with interaction testing and Chi-square test to assess whether the treatment effect was consistent across *post-hoc* subgroups. *P*-values of ≤ 0.05 were considered to indicate statistical significance. One-way ANOVA was used to analyze intergroup differences with a Bonferroni multiple comparison post-test. *P*-values <0.0167 were considered statistically significant.

## Results

### Baseline Characteristics

**Figure 1:**
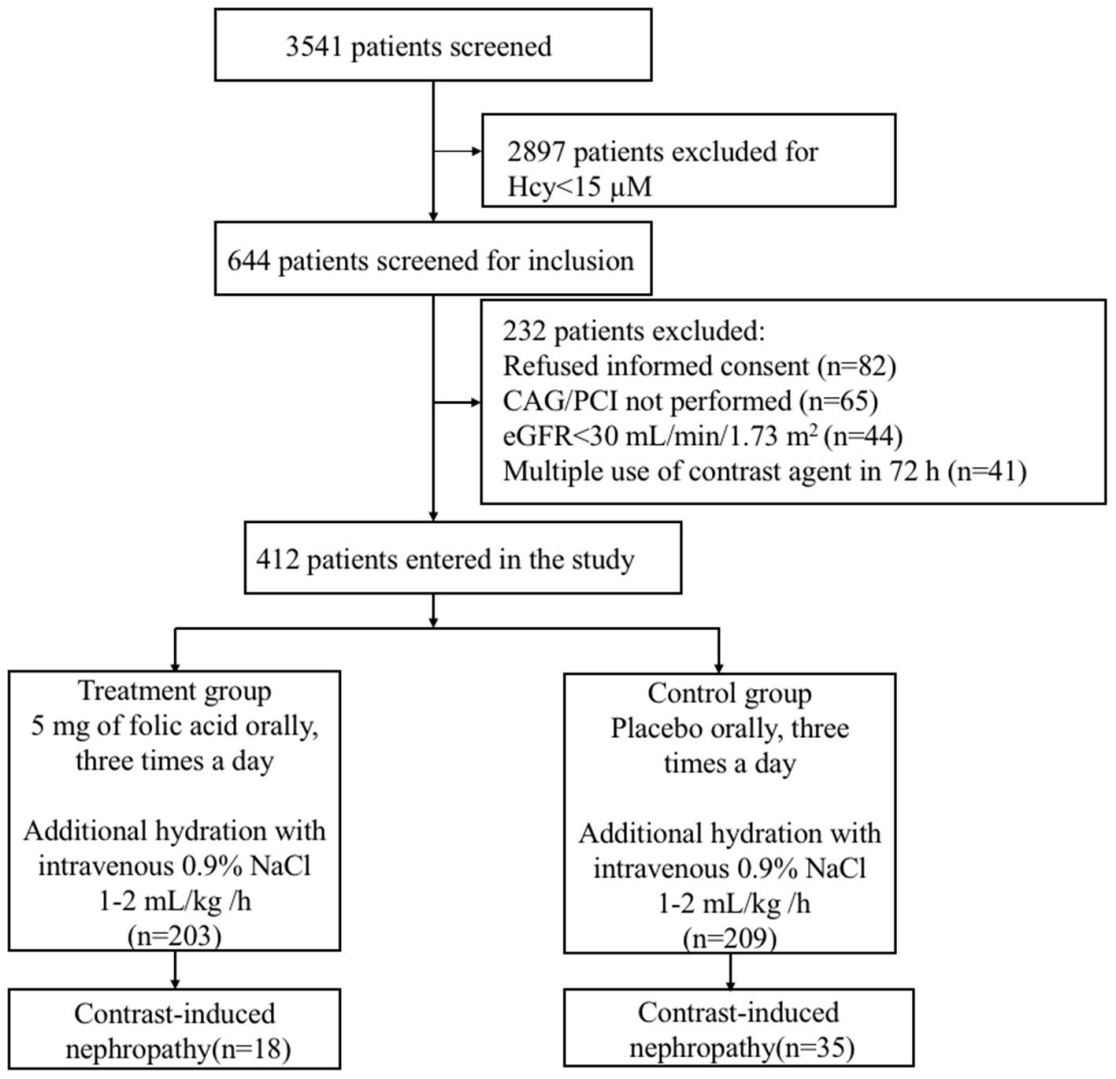
Flowchart of the study population of patients with hyperhomocysteinemia receiving folic acid and the control group. CAG, coronary arteriography; PCI, percutaneous coronary intervention; eGFR, estimated glomerular filtration rate.

Among the 3,541 consecutive patients who underwent CAG or PCI, the plasma Hcy concentration was ≥15 μM in 644 patients screened for inclusion (Figure 1). Ultimately, 412 patients (mean age: 65 ± 12 years, 268 male and 144 female) participated in the study, with 203 in the treatment group and 209 in the control group. All randomized patients who provided consent, constituting the full analysis set, were included according to ITT principles for the efficacy analyses. Baseline characteristics of the study population are presented in Table 1. The mean eGFR of all patients was 61 ± 19 mL/min/1.73 m^2^. There were similar distributions of clinical diagnosis in two groups. In terms of operations, 245 (59%) patients received CAG and 167 (41%) received PCI, with a mean contrast-material volume of 106.1 ± 49.8 mL. The mean pre- and post-operative intravenous hydration volumes were 672.4 ± 87.7 and 752.3 ± 116.8 mL, respectively. All stents are drug eluting stent. All randomly assigned patients received their allocated treatment (Figure 1).

**Table 1:** Baseline characteristics of patients with hyperhomocysteinemia receiving folic acid vs. a control group.

	**Treatment group**	**Control group**	***P*-value**
	**(*n* = 203)**	**(*n* = 209)**	
Age, years	66 ± 12	65 ± 12	0.293
Male, *n* (%)	138 (68)	130 (62)	0.219
BMI, kg/m^2^	24.9 ± 3.5	24.7 ± 3.2	0.378
Systolic blood pressure, mmHg	139.27 ± 20.21	138.46 ± 22.72	0.396
Diastolic blood pressure, mmHg	81.71 ± 13.31	79.58 ± 12.38	0.149
Heart rate, bpm	75.31 ± 12.25	74.82 ± 12.76	0.350
Hypertension, *n* (%)	158 (78)	152 (73)	0.153
Diabetes mellitus, *n* (%)	70 (34)	78 (37)	0.548
Hyperlipidemia, *n* (%)	72 (35)	69 (33)	0.600
CHF, *n* (%)	24 (12)	25 (12)	0.965
Anemia, *n* (%)	32 (16)	36 (17)	0.690
Current smoker, *n* (%)	86 (42)	86 (41)	0.802
Hemoglobin, g/L	131.7 ± 18.7	130.9 ± 18.7	0.588
Total cholesterol, mM	4.47 ± 1.17	4.53 ± 1.30	0.792
Triglyceride, mM	1.79 ± 1.59	1.56 ± 1.19	0.089
HDL-C, mM	1.02 ± 0.28	1.11 ± 0.28	0.001
LDL-C, mM	2.83 ± 1.03	2.88 ± 1.11	0.748
Fasting glucose, mM	5.81 ± 1.64	5.70 ± 1.61	0.390
HbA1C, %	6.11 ± 0.95	6.27 ± 1.30	0.708
BUN, μM	5.77 ± 1.96	5.41 ± 1.81	0.842
Scr, μM	99.02 ± 27.61	97.14 ± 21.78	0.441
eGFR, mL/min/1.73 m^2^	59 ± 19	60 ± 19	0.285
LVEF, %	55.17 ± 9.40	56.13 ± 7.34	0.271
ACEI/ARB, *n* (%)	115 (57)	114 (55)	0.667
Statins, *n* (%)	183 (90)	197 (94)	0.119
Diuretic, *n* (%)	86 (42)	86 (41)	0.802
Aspirin, *n* (%)	139 (68%)	150 (72%)	0.465
Clopidogrel, *n* (%)	94 (46%)	114 (55%)	0.094
Ticagrelor, *n* (%)	26 (13%)	28 (13%)	0.859
**Clinical diagnosis**			
ACS	56 (28%)	58 (28%)	0.970
CAD	76 (37%)	79 (28%)	0.940
NCHD	71 (35%)	72 (34%)	0.911
**Procedure**, ***n*** **(%)**			0.797
CAG	122 (60)	123 (59)	
PCI	81 (40)	86 (41)	
Duration of procedure, min	77.9 ± 52.2	83.6 ± 55.4	0.282
Contrast volume, mL	102.5 ± 48.2	109.7 ± 51.1	0.150
Prehydration, mL	672.9 ± 89.4	672.0 ± 86.0	0.512
Post-hydration, mL	760.1 ± 132.3	744.7 ± 101.8	0.368
Length of stay, day	9.76 ± 1.6	10.0 ± 2.0	0.133
Hospitalization cost,¥	27,464.9 ± 21,638.3	22,857 ± 22,083.9	0.033

*Categorical variables are presented as n (%). Continuous variables are presented as mean ± SD. BMI, body mass index; CHF, congestive heart failure; HDL-C, high-density lipoprotein cholesterol; LDL-C, low-density lipoprotein cholesterol; HbA1C, hemoglobin A1C; BUN, blood urea nitrogen; Scr, serum creatinine; eGFR, estimated glomerular filtration rate; LVEF, left ventricular ejection fraction; ACEI, angiotensin converting enzyme inhibitor; ARB, angiotensin receptor blocker; ACS, acute coronary syndrome; CAD, Chronic coronary artery disease; NCHD, Non-coronary heart disease; CAG, coronary arteriography; PCI, percutaneous coronary intervention*.

### Outcomes

#### Primary Endpoint

In total, 50 (12%) patients developed CIN after 48 h of CAG/PCI, including 16 (8%) in the treatment group and 34 (16%) in the control group (*P* = 0.009). Meanwhile, 53 (13%) patients developed CIN after 72 h of CAG/PCI, including 18 (9%) in the treatment group and 35 (17%) in the control group (*P* = 0.017). Regardless of the adopted standard, the incidence of CIN was significantly lower in the treatment group than in the control group.

As indicated in Table 2, the highest Scr concentrations were significantly higher within 48 h after the initiation of PCI than those at baseline in the two groups (treatment group: 107.42 ± 30.33 vs. 99.02 ± 27.61 μM, *P* = 0.0037; control group: 107.66 ± 24.56 vs. 97.14 ± 21.78 μM, *P* < 0.001). Otherwise, the highest Scr concentrations were significantly higher within 72 h after the initiation of PCI than those at baseline in the two groups (treatment group: 108.74 ± 30.60 vs. 99.02 ± 27.61 μM, *P* = 0.0009; control group: 110.86 ± 23.83 vs. 97.14 ± 21.78 μM, *P* < 0.001). The change in Scr concentration (Δ Scr) from pre-PCI baseline (0 h) to 72 h after PCI in the folic acid group was significantly lower than that in the control group (9.71 ± 13.77 vs. 13.73 ± 12.08 μM, *P* = 0.002). However, there was no significant difference in the Δ Scr within 48 h between two groups (8.39 ± 13.72 vs. 10.52 ± 13.12 μM, *P* = 0.108). The linear mixed model showed that folic acid decreased the Scr concentration (*P* < 0.0001 for group and group-time interaction).

**Table 2:** Change in serum creatinine concentration from pre-PCI baseline to 48 h and 72 h after the initiation of PCI in two groups.

	**Treatment group (*n* = 203)**	**Control group (*n* = 209)**	***P*-value**
Baseline Scr, μM	99.02 ± 27.61	97.14 ± 21.78	0.441
48 h max Scr, μM^a^	107.42 ± 30.33	107.66 ± 24.56	0.928
ΔScr (48 h max – baseline), μM	8.39 ± 13.72	10.52 ± 13.12	0.108
72 h max Scr, μM^a^	108.74 ± 30.60	110.86 ± 23.83	0.432
ΔScr (72 h max – baseline), μM	9.71 ± 13.77	13.73 ± 12.08	0.002

a *Highest serum creatinine (Scr) concentration within 48 or 72 h; ΔScr, change in serum creatinine concentration. All values are presented as mean ± SD*.

**Figure 2:**
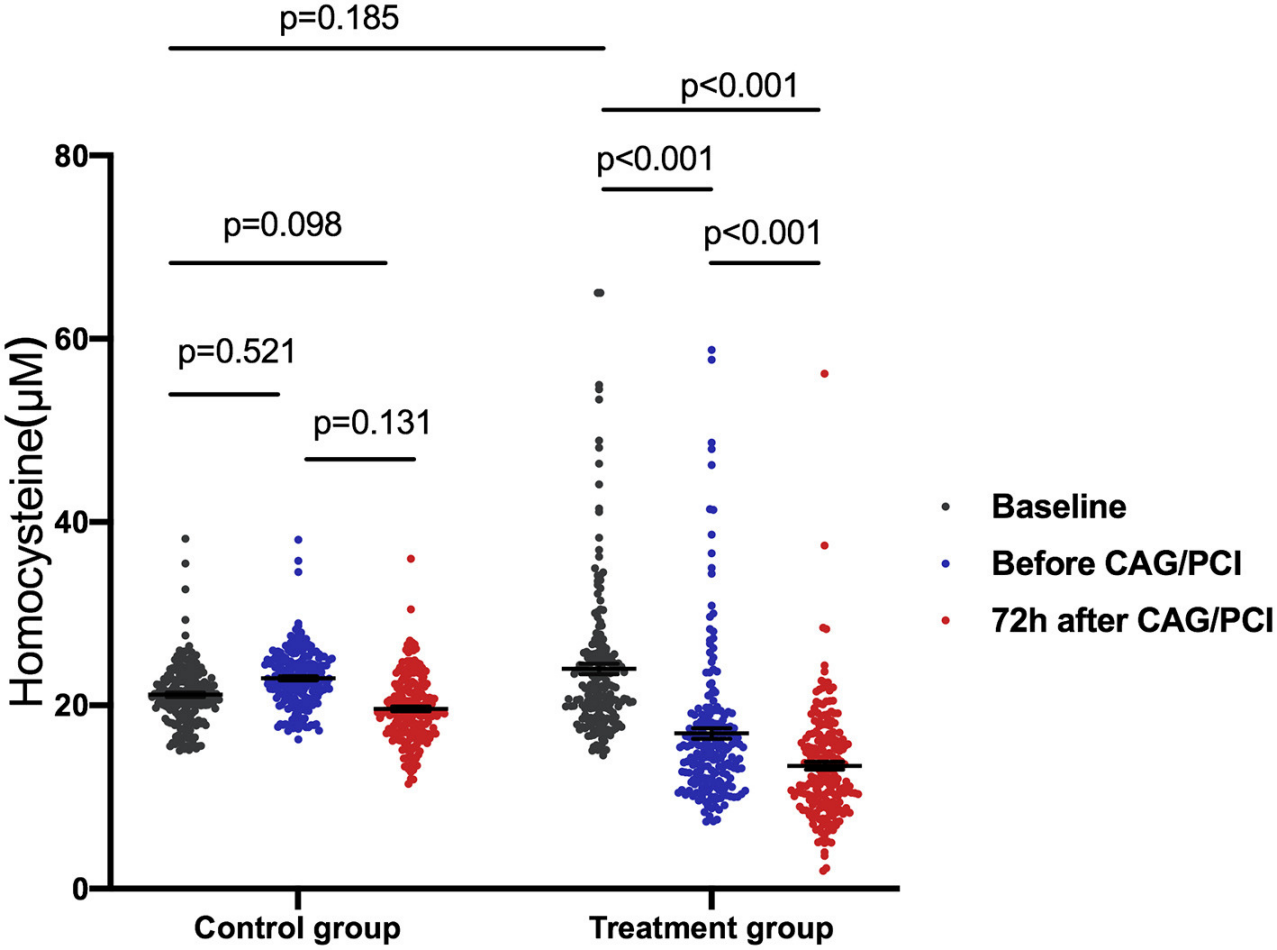
Homocysteine (Hcy) change over time in patients with hyperhomocysteinemia receiving folic acid and the control group. One-way ANOVA was used to analyze intergroup differences with a Bonferroni multiple comparison post-test. *P* < 0.0167 were considered statistically significant. The linear mixed model showed significant differences in the change in Hcy concentration between the two groups due to the interaction between the groups and time (*P* < 0.001). The folic acid group showed a faster decline. The average of the control group was 0.81 lower than that of the folic acid group at baseline, 6.02 higher than that of the folic acid group before procedure, and 9.05 higher than that at 72 h after procedure. ANOVA, analysis of variance; CAG, coronary arteriography; PCI, percutaneous coronary intervention.

In the treatment group, the baseline concentration of plasma Hcy was 23.99 ± 8.24 μM. After taking folic acid for a median duration of 6.0 (range, 5.0–8.0) days, the concentration of plasma Hcy decreased significantly to 16.94 ± 8.26 μM by the day before CAG/PCI (*P* < 0.001 vs. baseline). At 72 h after CAG/PCI, the concentration of plasma Hcy had further reduced to 13.41 ± 6.01 μM (*P* < 0.001 vs. baseline and vs. the day before CAG/PCI). In the control group, the baseline concentration of plasma Hcy (23.16 ± 3.30 μM) was similar to that of the treatment group (*P* = 0.19). Furthermore, the concentrations of plasma Hcy in the control group did not change significantly during the study (*P* > 0.05; Figure 2). The linear mixed model showed significant differences in change in the Hcy concentration between the two groups, due to the interaction between the groups and time, with the folic acid group showing a faster decline.

#### Secondary Outcomes

Table 3 shows the major adverse clinical events. There were no serious adverse events related to study treatment, and no death or hemodialysis occurred in either group. The rate of worsening heart failure events was lower, although not significantly so, in the treatment group (relative difference [RD] = 0.0138, 95% confidence interval [CI]: −0.0244 to −0.0532, *P* = 0.54). There was no statistical difference in the occurrence of bleeding and ICU admission rate between the two groups. No safety signal related to folic acid administration emerged during the study.

**Table 3:** Incidence of contrast-induced nephropathy and major adverse events in 72 h.

	**Treatment group**	**Control group**	**Risk difference**	***P-*value**
	**(*n* = 203)**	**(*n* = 209)**	**(95% CI)**	
CIN-48 h	16/203 (8.9%)	34/209 (16.7%)	0.0839 (0.0172 to 0.1505)	0.009
CIN-72 h	18/203 (8.9%)	35/209 (16.7%)	0.0788 (0.0105 to 0.1469)	0.017
Bleed	5/203 (2.5%)	5/209 (2.4%)	0.0007 (−0.0367 to −0.0389)	1.00
Worsening heart failure	4/203 (2%)	7/209 (3.3%)	0.0138 (−0.0244 to −0.0532)	0.54
All-cause mortality	0/203	0/209	–	1.00
Dialysis	0/203	0/209	–	1.00
Intensive care admission	2/203 (1.0%)	3/209 (1.4%)	0.0045 (−0.0265 to 0.036)	1.00

Univariate logistic regression analysis indicated that treatment with folic acid could lower the risk of CIN significantly (RD = 0.0788, 95%CI: 0.0105–0.1469, and *P* = 0.019). We further performed *post-hoc* subgroup analyses stratified by hypertension, diabetes mellitus, heart failure, age, sex, eGFR, and anemia with 72 h (Table 4). Administration of folic acid was a protective factor against CIN in patients with hypertension, diabetes mellitus, no heart failure, age>65 years, eGFR > 60 ml/min/1.73 m^2^, and no anemia, and those who were male (*P* < 0.05), except in patients with congestive heart failure (*P* = 0.299) or anemia (*P* = 0.34). The *P-*value of interaction testing for logistic regression in *post-hoc* subgroups was >0.05, except for sex.

**Table 4:** Occurrence of contrast-induced acute kidney injury in *post-hoc* subgroups.

	**Treatment**	**Control**	**Risk difference**	***P*-value**	***P-*value for**
	**group**	**group**	**(95% CI)**		**intervention**
**Hypertension**					0.712
Yes (*n* = 310)	15/158	26/126	0.1114 (0.0234 to 0.2033)	0.048	
No (*n* = 102)	3/45	9/57	0.0912 (−0.0578 to 0.2263)	0.156	
**Diabetes mellitus**					0.35
Yes (*n* = 148)	5/70	15/78	0.1209 (−0.001 to 0.238)	0.032	
No (*n* = 264)	13/123	20/131	0.047 (−0.0431 to 0.1355)	0.177	
**CHF**					0.927
Yes (*n* = 49)	3/24	6/25	0.115 (−0.1362 to 0.3491)	0.299	
No (*n* = 363)	15/179	29/184	0.0738 (0.0023 to 0.1453)	0.031	
**Age**					0.632
>65 years (*n* = 190)	10/123	12/67	0.0978 (−0.0059 to 0.221)	0.044	
≤ 65 (*n* = 222)	8/80	23/142	0.062 (−0.0455 to 0.1523)	0.201	
**Sex**					0.046
Male (*n* = 268)	8/138	23/130	0.119 (0.0369 to 0.2036)	0.201	
Female (*n* = 198)	10/65	12/79	0.0019 (−0.1242 to 0.1358)	0.754	
**eGFR**					0.967
>60 ml/min/1.73 m^2^ (*n* = 214)	9/104	20/110	0.0953 (−0.0042 to 0.193)	0.042	
≤ 60 ml/min/1.73 m^2^ (*n* = 198)	9/99	15/99	0.0606 (−0.0395 to 0.161)	0.191	
**Anemia**					0.804
Yes (*n* = 68)	5/32	9/26	0.1899 (−0.0549 to 0.4215)	0.34	
No (*n* = 344)	13/171	26/173	0.0743 (0.0026 to 0.1464)	0.03	

*CHF, chronic heart failure; eGFR, estimated glomerular filtration rate*.

## Discussion

Intravenous iodinated contrast is associated with a high risk for CIN, which may lead to increased morbidity, prolonged hospitalization, an increased risk of complications, a potential need for dialysis, and an increased mortality rate (5). Therefore, it is crucial to consider strategies for the prevention of CIN. Previous studies indicated HHcy as a risk factor for CIN (12, 13), and that folic acid can attenuate CIN in rats (16). In the present study, of 412 consecutive patients with HHcy undergoing coronary catheterization, we confirmed that perioperative treatment with folic acid could lead to a statistically significant reduction of plasma Hcy concentration and was associated with a lower incidence of CIN compared to those in patients treated with placebo. Logistic regression analysis demonstrated that folic acid was a protective factor against CIN. At the same time, 50 and 53 patients developed CIN within 48 and 72 h, respectively; thus it is necessary to extend the time of creatinine detection in clinical practice.

Many clinical trials have been conducted to test the abilities of various substances to reduce the incidence of CIN, such as acetylcysteine, sodium bicarbonate, statins, and furosemide. However, such attempts have yielded contradictory results (21–25), and the best strategy remains unclear. KDIGO (Kidney Disease: Improving Global Outcomes) published the first international and interdisciplinary clinical practice guideline on acute kidney injury, recommending intravenous prehydration with 0.9% NaCl as the basic strategy in patients at an increased risk for CIN (26). Therefore, in the present study, all participants received prophylactic hydration with intravenous 0.9% NaCl (1–2 mL/kg/h) at 6 h before and after contrast administration. Nonetheless, >12% of patients still developed CIN after catheterization. According to previous reports, the incidence of CIN is ~2% in patients with normal renal function and without other risk factors; however, the incidence can increase to 9–40% in high-risk patients (27). The most important risk factor for CIN is pre-existing renal insufficiency (28). The mean eGFR in the present study was <60 mL/min/1.73 m^2^, indicating that many participants had pre-existing renal insufficiency. Besides, many patients had other risk factors for CIN, such as old age, anemia, diabetes, and heart failure. This may explain why the incidence of CIN in the present study was relatively high, despite adequate perioperative hydration. Severe CIN may require hemodialysis to remove the contrast media from the blood (29), which is why we excluded patients with eGFR <30 mL/min/1.73 m^2^. Perhaps we excluded patients who used contrast agents multiple times in a short period of time, which means that some patients with complex conditions were not included. This may explain why there were less serious complications occur serious complications in the present study.

In order to reduce the risk of CIN, it is extremely important to identify patients at a high risk of CIN and to develop new pretreatment strategies. The pathogenesis of CIN is closely related to endothelial dysfunction and cellular toxicity induced by the contrast agent, as well as to tubular apoptosis resulting from hypoxic damage and reactive oxygen species (26). As elevated Hcy concentrations can induce endothelial dysfunction, apoptosis, oxidative stress, and thrombosis (9, 11), it follows that there is a potential link between Hcy and the risk of CIN; however, such a hypothesis has not been extensively evaluated. As we have previously demonstrated (12) that plasma Hcy concentration is an independent biomarker for predicting CIN, and as Barbieri et al. (13) demonstrated a statistically significant relationship between plasma Hcy concentration and the risk of CIN, reduction of the plasma Hcy concentration may reduce the risk of CIN.

Folic acid is an essential co-factor in Hcy metabolism (30); it exerts antioxidative and anti-apoptotic effects and improves endothelial functional properties (31). The administration of folic acid attenuated CIN statistically significantly in diabetic rats, the mechanism of which may be related to the inhibition of oxidative stress (16, 32). In clinical practice, folic acid is used in the management of many diseases, including diabetes mellitus, hypertension, and cardiovascular disorders (14, 15, 31). A meta-analysis of the efficacy of folic acid supplementation in stroke prevention demonstrated that folic acid supplementation can effectively reduce the risk of stroke in primary prevention (33). Xu et al. (34) found that enalapril-folic acid therapy, compared with enalapril alone, can significantly delay the progression of CKD among patients with mild-to-moderate disease.

In the present study, folic acid administration was first demonstrated to reduce both the plasma Hcy level and incidence of CIN statistically significantly after coronary catheterization. Subgroup analyses further confirmed that administration of folic acid was an independent protective factor against CIN, except in patients with congestive heart failure or anemia. However, as the number of participants with congestive heart failure (*n* = 49) or anemia (*n* = 68) was relatively small in our study, the effectiveness of folic acid in protecting such patients against CIN needs to be verified. Based on its potential benefits and few side effects, we recommend perioperative administration of folic acid to attenuate CIN in patients with HHcy undergoing coronary catheterization.

Several limitations of the present study should be noted. First, this was a single-center study. Analyses involving different surgeons and using therapeutic strategies may lead to different results. Second, the overall sample size was relatively small; clinical trials with larger samples should be conducted for verification of our results. Third, this study included only HHcy patients. Potential preventive effects of folic acid on CIN may arise not only from a reduction in Hcy concentration, but also because of its antioxidative and anti-apoptotic properties. Therefore, research into the effect of folic acid in non-HHcy patients is also warranted. Finally, as the pathophysiological link between folic acid and CIN remains unclear, further studies will be required to elucidate the potential molecular mechanism underlying its action.

In conclusion, results from the current study suggest that perioperative administration of folic acid is associated with a statistically significant reduction in the incidence of CIN after coronary catheterization in patients with HHcy. More studies on the potential molecular mechanism should be conducted to uncover the exact role of folic acid in the protection against CIN.

## Data Availability Statement

The raw data supporting the conclusions of this article will be made available by the authors, without undue reservation.

## Ethics Statement

The studies involving human participants were reviewed and approved by the Third Affiliated Hospital, Sun Yat-sen University. The patients/participants provided their written informed consent to participate in this study.

## Author Contributions

SL and HP contributed to the study concepts, study design, and supervision. LP and XS drafted the manuscript and contributed to patient enrollment and follow-up. FT and ZL participated in the interpretation of data and statistical analyses. YL and BW were responsible for data collection. LC helped in revising the manuscript. All authors were involved in reporting the results of this study and approved the final version of the submitted manuscript.

## Funding

This work was supported by the National Natural Science Foundation of China (Grant Numbers 81900320 and 81470955), the Medical Science and Technology Research Project of Guangdong Province (Grant Number C2019107), the basic research funding of Sun Yat-sen University (Grant Number 19ykpy40), the Guangzhou Science and Technology Project (Grant Number 201807010037), and the National Natural Science Foundation of Guangdong Province (Grant Number 2017A030313714).

## Conflict of Interest

The authors declare that the research was conducted in the absence of any commercial or financial relationships that could be construed as a potential conflict of interest.

## Publisher's Note

All claims expressed in this article are solely those of the authors and do not necessarily represent those of their affiliated organizations, or those of the publisher, the editors and the reviewers. Any product that may be evaluated in this article, or claim that may be made by its manufacturer, is not guaranteed or endorsed by the publisher.

## Abbreviations

CAG: coronary arteriography
CIN: contrast-induced nephropathy
eGFR: estimated glomerular filtration rate
Hcy: homocysteine
HHcy: hyperhomocysteinemia
ICU: intensive care unit
PCI: percutaneous coronary intervention
Scr: serum creatinine.
